# Vitamin C: From Self-Sufficiency to Dietary Dependence in the Framework of Its Biological Functions and Medical Implications

**DOI:** 10.3390/life15020238

**Published:** 2025-02-05

**Authors:** Andrei Cristian Grădinaru, Setalia Popa

**Affiliations:** 1 Faculty of Veterinary Medicine, “Ion Ionescu de la Brad” University of Life Sciences, 3 M. Sadoveanu Alley, 700490 Iasi, Romania; 2 Faculty of Medicine, “Grigore T. Popa” University of Medicine and Pharmacy, 700115 Iasi, Romania

**Keywords:** *GULO* gene, pseudogenization, anti-oxidant, enzymatic cofactor

## Abstract

Vitamin C is an organic compound biosynthesized in plants and most vertebrates. Since its discovery, the benefits of vitamin C use in the cure and prevention of various pathologies have been frequently reported, including its anti-oxidant, anti-inflammatory, anticoagulant, and immune modulatory properties. Vitamin C plays an important role in collagen synthesis and subsequent scurvy prevention. It is also required in vivo as a cofactor for enzymes involved in carnitine and catecholamine norepinephrine biosynthesis, peptide amidation, and tyrosine catabolism. Moreover, as an enzymatic cofactor, vitamin C is involved in processes of gene transcription and epigenetic regulation. The absence of the synthesis of L-gulono-1,4-lactone oxidase, a key enzyme in the pathway of vitamin C synthesis, is an inborn metabolism error in some fishes and several bird and mammalian species, including humans and non-human primates; it is caused by various changes in the structure of the original *GULO* gene, making these affected species dependent on external sources of vitamin C. The evolutionary cause of *GULO* gene pseudogenization remains controversial, as either dietary supplementation or neutral selection is evoked. An evolutionary improvement in the control of redox homeostasis was also considered, as potentially toxic H_2_O_2_ is generated as a byproduct in the vitamin C biosynthesis pathway. The inactivation of the *GULO* gene and the subsequent reliance on dietary vitamin C may have broader implications for aging and age-related diseases, as one of the most important actions of vitamin C is as an anti-oxidant. Therefore, an important aim for medical professionals regarding human and animal health should be establishing vitamin C homeostasis in species that are unable to synthesize it themselves, preventing pathologies such as cardiovascular diseases, cognitive decline, and even cancer.

## 1. Introduction

Vitamin C, also known as L-ascorbic acid or ascorbate (ASC), is an organic compound (C_6_H_8_O_6_) chemically named 2-oxo-L-threo-hexono-1,4-lactone-2,3-enediol. It was first isolated in 1928 by Albert Szent-Györgyi, using lemons, pepper, and adrenal glands as extraction substrates. It was initially called “ignose” and later renamed “hexuronic acid”. In 1932, vitamin C was isolated again by Charles Glen King, who described it as being the same substance as the already known “hexuronic acid”. In 1933, Norman Haworth established the chemical structure of vitamin C [1,2]. Considering its evident ability to prevent scurvy, “hexuronic acid” was renamed “ascorbic acid” in 1963 by Albert Szent-Györgyi and Norman Haworth [3] (in Latin, “scorbutus”—hence, “a-scorbutus”). The oxidized form, L-dehydroascorbate (DHA), is usually included in the general group of vitamin C, as it can be easily converted into ASC in the body. In addition to L-ascorbic acid, other optical isomers of vitamin C, such as erythorbic acid (also named isoascorbic acid or D-araboascorbic acid) and D-ascorbic acid, are also included in the general group of vitamin C, although their retention at the tissue level is decreased and they are rapidly excreted from the body [4]. The most well-known name of “vitamin C” was coined after the names “fat-soluble vitamin A” and “water-soluble vitamin B” had already been introduced [4,5].

High levels of vitamin C are found in citrus fruits, cabbage-type vegetables, dark green vegetables, cantaloupe, lettuce, tomatoes, potatoes, papayas, and mangoes in quantities of 10–100 mg/100 g, which are much higher than other vitamins [5]. It is found in high concentrations in many plant organs, but particularly in the leaves and fruits. Vitamin C is a carbon source in plants for intercellular CO_2_ and metabolites, such as L-tartaric, L-threonic, L-glyceric, and oxalic acids; its involvement in recycling carbon into products via triose and hexose phosphate metabolism is well recognized [6]. It is essential for plant growth and development through the ascorbate-dependent limitation of reactive oxygen species (ROS) accumulation (especially in chloroplasts), NO production modulation in the mechanism of flowering control, and as a cofactor in various reactions—for example, in the stabilization of 2-oxoglutarate-dependent dioxygenases that are necessary for phytohormones synthesis [7]. However, when discussing the biological significance of vitamin C in plants, the Foyer–Asada–Halliwell cycle, also known as the ascorbate–glutathione cycle, is worth mentioning [8,9]. This cycle is now accepted as essential for plant survival under various environmental challenges, ensuring cellular protection against oxidative damage, subsequently leading to optimal plant growth and development [9,10]. With ascorbate playing the central role in ROS (particularly H_2_O_2_) scavenging, the efficiency of this cycle obviously depends on ascorbate enzymatic regeneration from its mono-oxidized form and/or its fully oxidized form. Nevertheless, glutathione, as another important anti-oxidant that works in conjunction with ascorbate in this cycle, also needs enzymatic regeneration for a continuous supply of reduced glutathione, therefore ensuring the efficient functionality of the cycle [11,12].

Despite its abundance in vegetable substrates, vitamin C is easily destroyed by light, heat, and oxygen [13]. Wild plants contain higher levels of vitamin C than domestic ones, in addition to other anti-oxidants (e.g., β-carotene, lycopene, or vitamin E). Candidate genes associated with increased contents of vitamin C are used as markers in plant breeding programs to increase their anti-oxidant contents [14].

Since its discovery, the benefits of vitamin C use in the cure and prevention of various pathologies have been frequently reported [15,16,17]. Some fishes and several bird and mammalian species, including humans and non-human primates, have lost their ability to synthesize vitamin C, becoming dependent on exogenous sources [13,18,19,20,21,22,23]. This inability is genetically caused by mutations that occurred in the structure of the ancestral *GULO* gene, leading to its inactivation. The inactivation of the *GULO* gene led to a subsequent lack of the synthesis of L-gulono-1,4-lactone oxidase (L-gulono-γ-lactone oxidase, Gulo) (EC 1.1.3.8). This was a long-lasting result of a complex evolutionary process hypothesized to involve several mechanisms, such as neutral mutation and neutral evolution; environmental changes through the atmospheric alleviation of hyperoxia conditions; or safety measures in the control of redox homeostasis of organisms, as byproducts that would result from endogenous vitamin C biosynthesis are harmful at the cellular level [5,24]. Radiation exposures or a retrovirus were also hypothesized as possible causes for this genetic change [25].

This study focuses on important elements regarding the physiological role of vitamin C and its medical implications, general pathways of biosynthesis, evolutionary loss of certain species to synthesize it, and mechanisms of its transport at the cellular level.

## 2. Normal Vitamin C Levels in the Body and Its Daily Requirements

Vitamin C is a vital nutrient that plays a significant role in maintaining human and animal health. As a member of the water-soluble vitamin family, vitamin C cannot be stored in the body; therefore, it is essential to obtain it regularly through diet or supplements [26,27]. In the medical field, the importance of vitamin C in supporting various bodily functions and promoting overall well-being cannot be overstated.

The human body contains ~1.5 g vitamin C (20 mg/kg) [4] and loses ~3% of its vitamin C content per day [28]. Plasma and leukocyte levels of vitamin C reflect the amount absorbed from the digestive tract, making them important to consider when evaluating the bioavailability of vitamin C [29]. McCall et al. [30] considered vitamin C deficiency as <11 µmol/L, suboptimal plasma vitamin C levels as ≥11–28 µmol/L, and adequate levels of plasma vitamin C as >28 µmol/L. Other reports showed that hypovitaminosis C usually occurs when the plasma concentration of vitamin C drops below 23 μmol/L, affecting between 5 and 10% of adults in the industrialized world [21,31], although larger shares of 44% and 47% were reported for populations investigated in Scotland (Glasgow) in 1992 and Canada (Toronto) between 2004 and 2008 [32]. Usually, higher concentrations of ascorbic acid are quantified in tissues than in plasma [33]. Additional values for normal vitamin C concentration in various human tissues, human blood plasma, and certain cells, as well as in some human fluids, are presented in Table 1.

A vitamin C daily intake of ~80 mg is recommended to ensure a saturated body pool (90 mg for men and 75 mg for women). This daily intake has to be increased by an additional 35 mg for smokers (to an upper limit of 140 mg per day), considering their increased vitamin C needs to inactivate oxidants from tobacco smoke. An additional 5–10 mg of vitamin C is recommended in pregnant women, with +25 mg for lactating mothers [4]. Doses of ≥500 mg/day are recommended following surgery in order to shorten the wound healing time [21]. However, a tolerable daily upper intake of 2000 mg vitamin C was established by the Food and Nutrition Board in 2000 [15,21,34,35,36,37,38,39,40].

An increased demand for vitamin C in people living with obesity was also found for individuals with prediabetes and type 2 diabetes mellitus [41]. Some drug interactions make vitamin C unavailable, whether medicinally or through food. For example, there is a pharmacological association between vitamin C and aspirin (an antipyretic, analgesic, and antirheumatic medicine) when the latter is taken first, with the presence of aspirin seemingly decreasing the absorption of vitamin C in the human body; however, taking vitamin C first does not affect the absorption of aspirin [42].

Macaques and squirrel monkeys need a daily vitamin C intake of 23–35 mg per kg of body weight (corresponding to 1610–2450 mg in a 70 kg man); baboons require 10 mg per kg (corresponding to 700 mg daily in a man); and guinea pigs need 10 to 16 mg per kg (corresponding to 700–1120 mg in a man) [43]. These data show large differences in vitamin C requirements between humans and other species that also do not synthesize vitamin C by themselves; therefore, an extrapolation of data obtained in these animals is not recommended regarding humans. In animals that synthesize endogenous vitamin C, requirements for exogenous vitamin C are increasingly different from those species without synthesis capacity. However, the synthesis rates of vitamin C (expressed in mg/kg/day) for applicable species can differ widely (see [43]).

Hypervitaminosis C is the condition that results from an excessive uptake of vitamin C over a long period of time; it includes gastrointestinal distress, symptoms of diarrhea and abdominal bloating (in single oral doses of 5–10 g or greater than 2 g daily, with symptoms disappearing within 1–2 weeks), overabsorption of iron, hyperoxalemia and calcium oxalate stones in patients with renal issues (healthy individuals can also produce excessive oxalate at doses greater than 1 g daily as a result of vitamin C conversion to oxalate during the process of urine excretion), and hemolysis (in patients with glucose-6-phosphate dehydrogenase deficiency) [13,27,38].

## 3. Debates on Vitamin C Regarding Health and Disease

### 3.1. Vitamin C Is Crucial for Supporting the Immune System

Vitamin C enhances the function of various immune cells and promotes the production of antibodies, which are essential for fighting off infections and illnesses. Adequate intake of vitamin C has been linked to reduced duration and severity of common colds and can help the body mount a stronger defense against pathogens [44].

The four main mechanisms through which vitamin C supports the immune system are as follows: (i) Increasing the production of white blood cells [45], which are the key players in immune response, meaning the body is better equipped to fight off infections. Moreover, vitamin C enhances phagocytosis by enhancing the ability of white blood cells to engulf and destroy foreign invaders. Vitamin C accumulation in neutrophils by up to 30-fold was reported, which not only supported neutrophils in their fight against bacteria, but also protected them from the resulting ROS damage [46,47]. (ii) Enhancing the production of interferon [48], which is a protein that plays a crucial role in antiviral defense, helping prevent the spread of viruses and limiting their replication (antiviral effects). (iii) Enhancing the iron absorption [49] from plant-based sources, known as non-heme iron [50]. This is particularly important for individuals following vegetarian or vegan diets, as iron deficiency is a common concern in these populations. By improving iron absorption, vitamin C helps to prevent and alleviate iron-deficiency anemia, a condition characterized by fatigue, weakness, and impaired cognitive function. This process of vitamin C facilitating absorption from the intestines is important because iron is crucial for the production of hemoglobin, a protein in red blood cells that carries oxygen to tissues, while adequate oxygen delivery is essential for optimal immune function and energy production. Despite vitamin C alone not directly increasing the hemoglobin levels, by improving iron absorption, vitamin C indirectly supports hemoglobin production [50,51,52]. (iv) Modulating cytokines production [53], signaling molecules that regulate immune responses.

### 3.2. Anti-Oxidant Properties of Vitamin C and the Reverse of Pro-Oxidant Action

One of the primary roles of vitamin C is provided by its potent anti-oxidant properties. As an anti-oxidant, vitamin C helps in neutralizing free radicals in the body, which are unstable molecules that can cause cellular damage and contribute to the development of chronic diseases, such as cancer and heart disease [54].

Generally, ROS are constituted by free radicals, such as the superoxide anion (O_2−_^•^), hydroxyl radical (OH^•^), peroxyl radical (RO_2_^•^), alkoxyl radical (RO^•^), hydroperoxyl radical (HO_2_^•^), and non-radical derivatives of oxygen, such as hydrogen peroxide (H_2_O_2_), hypochlorous acid (HOCl), ozone (O_3_), singlet oxygen (^1^O_2_), and the peroxynitrite anion (ONOO^−^). These metabolites of oxygen endanger cell survival, adversely modifying functions and mechanisms. Their excess is highly toxic for cells, inducing lipid peroxidation, protein and DNA damage through their oxidation, and depletion of ATP production (increased ROS levels correspond to decreased ATP levels), with final cell apoptosis and death, as well as structural tissue damage [55,56,57,58,59].

The effects of oxidative damage can be limited in a wide range of stressors through the action of complex anti-oxidant systems. In addition to vitamin C, these systems include enzymes such as superoxide dismutase (SOD), glutathione peroxidases (GPxs), and catalase, as well as glutathione, α-tocopherol (vitamin E), ubiquinol, and β-carotene [58]. Vitamin C is involved in the vitamin E anti-oxidant system through re-converting oxidized forms of α-tocopherol to α-tocopherol (by reducing the α-tocopheroxyl radical) [20,58,60], thus minimizing the pro-oxidant action of α-tocopherol when other anti-oxidants are missing (the co-anti-oxidant action of vitamin C) [35]. However, oxidative damage associated with subclinical ascorbic acid deficiency occurred in spite of adequate levels of other anti-oxidants, including α-tocopherol, glutathione, superoxide dismutase, catalase, and glutathione peroxidase [61]. Although peroxiredoxins (Prxs), thioredoxins (Trxs), and glutaredoxins (Grxs) are not direct anti-oxidants like vitamin C, they are crucial anti-oxidant enzymes that work together in maintaining redox balance within cells, contributing to the overall anti-oxidant defense system. They are recognized as key players in maintaining cellular redox homeostasis by effectively reducing oxidized molecules and creating an environment where vitamin C can function properly [62,63,64,65,66,67].

Vitamin C is a major cellular anti-oxidant, containing a highly reactive “ene-diol” group that transforms a relatively inactive sugar to a powerful reducing agent in aqueous solution (unlike glucose, although vitamin C is a water-soluble glucose derivative) [68].

**Figure 1:**
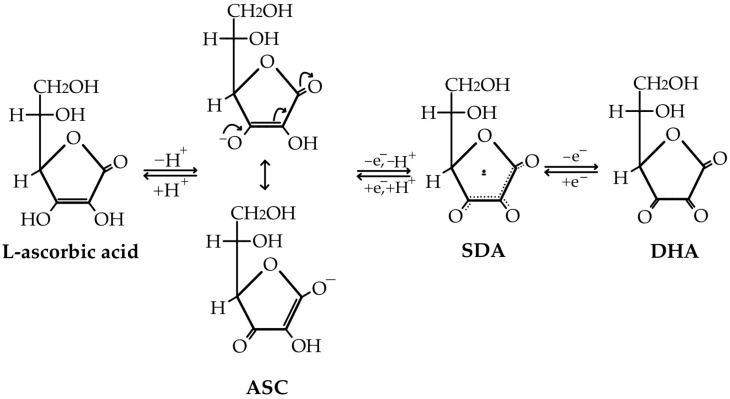
L-Ascorbic acid and the three redox states during its metabolism: **ASC**—ascorbate; **SDA**—semidehydroascorbate; **DHA**—dehydroascorbate (adapted from [5,20,69,70,71]).

Vitamin C has three redox states in organisms: (i) *ascorbate* (**ASC**; the fully reduced form), (ii) *semidehydroascorbate* (**SDA**; the mono-oxidized form, which is more stable and much less reactive and is also named as monodehydroascorbate (MDHA) or the ascorbyl radical), and (iii) *dehydroascorbate* (**DHA**; the fully oxidized form) (Figure 1) [5].

At a physiological pH (~7.4), most vitamin C circulates as the ascorbate monoanion resulting from hydrogen ion loss by the ascorbic acid molecule. Although the pKa of ascorbic acid does not directly determine its oxidation state, its lower value (~4.17) defines the dissociation state or how readily it loses a hydrogen ion (H^+^) to become deprotonated [33,72].

Alkaline pH, exposure to light and heat, and the presence of oxygen, water, or some metals (Cu, Fe) causes the oxidation of ascorbic acid through the dehydrogenation of the two hydroxyl groups at C_2_ and C_3_, with the formation of DHA [73]. This is a reversible reaction and represents the essence of vitamin C’s involvement in the body’s redox mechanism [69].

**Figure 2:**
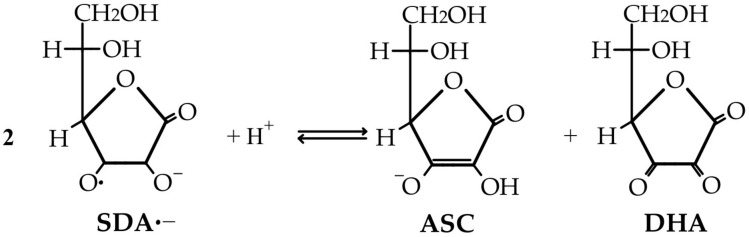
Disproportionation of **SDA** (semidehydroascorbate) when obtaining **ASC** (ascorbate) and **DHA** (dehydroascorbate) (adapted from [75]).

SDA may or may not be an immediate product of ASC oxidation when DHA is obtained [74]. However, the fulfillment of the anti-oxidant and cofactor functions of ASC leads to the production of SDA, which will underlie the regeneration of ASC and DHA through the spontaneous decay of this non-reactive free radical. In this regard, although SDA is relatively stable, it can undergo disproportionation, a reaction where two radicals combine to form one molecule of ASC and another of DHA (Figure 2). This reaction is very important in maintaining the redox balance within the cell and ensuring the continuous availability of ascorbate for anti-oxidant defense [74,75].

**Figure 3:**
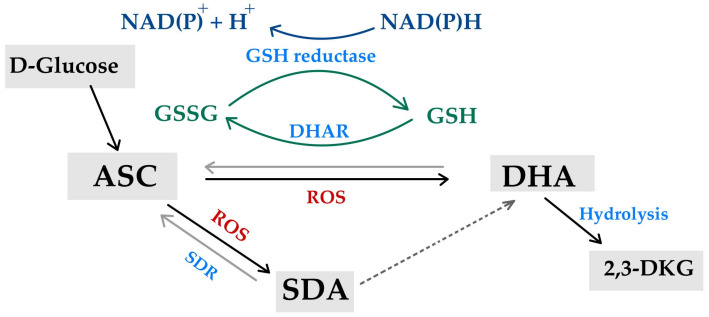
The anti-oxidant action of ascorbate. **ASC**—ascorbate; **SDA**—semidehydroascorbate; **DHA**—dehydroascorbate; **2,3-DKG**—2,3-diketo-L-gulonate; **GSH**—glutathione reduced; **GSSG**—glutathione disulfide (oxidized); **ROS**—reactive oxygen species; **SDR**—semidehydroascorbate reductase; **DHAR**—dehydroascorbate reductase; **GSH reductase**—glutathione reductase; **NAD(P)**—nicotinamide adenine dinucleotide phosphate; **NAD(P)H**—nicotinamide adenine dinucleotide phosphate (adapted from [79,80]).

**Figure 4:**
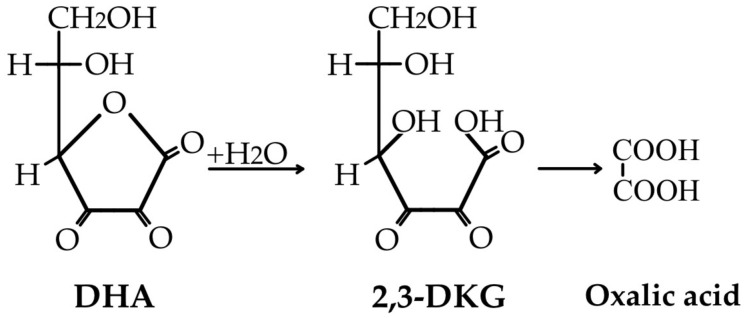
Irreversible DHA (dehydroascorbate) hydrolysis to 2,3-DKG (2,3-diketo-L-gulonate) and further oxidation of 2,3-DKG to oxalate (adapted from [5,69]).

The ascorbate’s direct anti-oxidant action is based on the stability of SDA, an intermediate product in ASC’s ROS reduction pathway, which can then be further oxidized to DHA or disproportionated back to ascorbate [74,76]. When considering the fate of SDA, it is notable that in addition to its further oxidation to DHA, it directly reduces to ASC in a one-electron step, with a possible immixture of semidehydroascorbate reductase (SDR) to the process of ASC regeneration (as was believed to be the case in the 1980s) [77]. The unstable DHA is now (i) either non-enzymatically reduced back to ascorbate in a recycling process that is slowly performed by glutathione or enzymatically catalyzed by NADPH-dependent reductases (Figure 3), (ii) or irreversibly lost through a hydrolysis reaction to 2,3-diketo-L-gulonate (2,3-DKG) (Figure 4). Once formed, 2,3-DKG can either be decarboxylated, with the products entering the pentose phosphate shunt through a couple of reactions, or spontaneously or enzymatically decomposed to L-erythrulose (ERY) and oxalic acid [4]. Smaller molecules resulting from 2,3-DKG oxidation (such as oxalic acid) are urinary excreted [5]. In the presence of H_2_O_2_, 2,3-DKG degradation can result in L-threonic acid and oxalic acid, with a transient five-carbon intermediate (3,4,5-trihydroxy-2-ketopentanoate) [27,78].

Vitamin C and glutathione peroxidase (GPx) work together to protect cells from oxidative damage. Vitamin C helps in maintaining the reduced glutathione state, ensuring the continuous function of GPx. This synergistic relationship contributes to the overall anti-oxidant defense system of the body. When ROS such as hydrogen peroxide (H_2_O_2_) are generated as byproducts of cellular metabolism and environmental factors, GPx intervenes in an enzymatic neutralization of H_2_O_2_ by reducing it to water, using reduced glutathione (GSH) as a cofactor. Although GSH is oxidized to oxidized glutathione (GSSG), its regeneration is directly made by glutathione reductase (GSH reductase) and NADPH, as an electron donor used to reduce GSSG back to GSH. However, the primary function of ASC is not that of GSH regeneration from GSSG, but it indirectly supports this process by maintaining the pool of reduced anti-oxidants within the cell [81].

A similar mechanism of ROS (particularly H_2_O_2_) scavenging through the ASC and glutathione action is described in plants in a crucial metabolic pathway named the Foyer–Asada–Halliwell cycle. A brief reference of this cycle was made in the introduction, when the biological significance of ascorbic acid was discussed. In this cycle, the powerful anti-oxidant action of ASC is conditioned by its use as an electron donor in the reduction of H_2_O_2_ in a reaction enzymatically catalyzed by ascorbate peroxidase (APx). As ASC directly reacts with and neutralizes ROS such as H_2_O_2_ through the action of APx, its consumption becomes an existential problem in this cycle. Therefore, its regeneration through SDR (that reduces SDA back to ASC) and DHAR (that reduces DHA back to ASC) is vital for maintaining the cycle’s efficiency [8,12,82,83]. Furthermore, glutathione reductase (GSH reductase) reduces oxidized glutathione (GGSG) back to its reduced form (GSH) using NADPH as an electron donor, ensuring the continuous supply of reduced glutathione (GSH) as an important anti-oxidant coworker [11,84].

**Figure 5:**
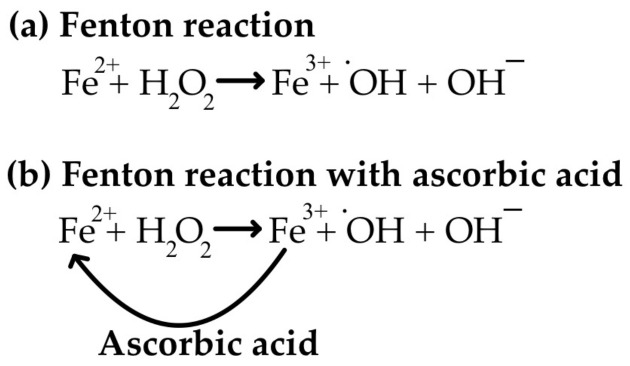
The pro-oxidant action of vitamin C by facilitating the generation of reactive oxygen species (ROS) as a result of Fe^3+^ to Fe^2+^ recycling (adapted from [88,89,90,91]).

While vitamin C is a potent anti-oxidant, excessive intake can paradoxically lead to increased oxidative stress [85]. An apparent contradiction between its anti-oxidant and pro-oxidant functions may be provided by ascorbate participation in the Fenton reaction, a process that generates highly reactive hydroxyl radicals. Ascorbate can reduce ferric ions (Fe^3+^) to ferrous ions (Fe^2+^), which can then react with hydrogen peroxide to form hydroxyl radicals (Figure 5), further leading to oxidative damage translated into the damage of cellular components, including DNA, proteins, and lipids [35,70,86,87,88,89,90,91,92]. However, ascorbate can also scavenge these hydroxyl radicals, limiting their damaging effects [92]. The pro-oxidant effects of vitamin C are typically observed at very high doses and are not a concern for most individuals. It is important to maintain a balanced intake of vitamin C and avoid excessive supplementation without medical supervision [93]. Halliwell [94] discussed ascorbate pro-oxidant effects in vitro, with limited evidence for occurrence in vivo. This is justified by the fact that the Fenton reaction requires both free ferrous irons (Fe^2+^) and hydrogen peroxide; however, in vivo redox-active iron cells are tightly regulated and sequestered within proteins such as ferritin and transferrin, minimizing the availability of free iron for Fenton reactions. While typical oral vitamin C supplementation does not significantly increase the risk of Fenton reaction in healthy individuals, conditions that disrupt iron homeostasis or increase free iron availability are more likely to promote Fenton-mediated oxidative damage. Notable conditions that favor Fenton reaction are as follows: (i) hemochromatosis, when iron overload occurs, significantly increasing the risk of Fenton-mediated oxidative damage; (ii) inflammation, tissue injury, and certain diseases that can disrupt iron homeostasis, leading to free iron increase and the potential for Fenton reactions; and (iii) protein deficiency, including conditions that impair protein synthesis or function, affecting iron-binding proteins such as ferritin and transferrin, thus increasing the availability of free iron [95,96].

In conclusion, ascorbate is an essential nutrient with potent anti-oxidant properties revealed in various clinical situations. For example, when taken in large doses (intravenous sodium ascorbate, 150 g per 40 kg over 7 h), vitamin C was effective in treating sepsis, with dramatic improvements in clinical status [97]. ROS formation within chondrocytes was included as a critical step in osteoarthritis pathogenesis, which is a reason that vitamin C’s anti-oxidant properties justified its use in osteoarthritis prevention and therapy [28,98]. The use of vitamin C in the treatment of stress-related diseases, such as depression and anxiety, is also possible because of its anti-oxidant neuroprotective effects, to which the modulation of monoaminergic and glutamatergic, dopaminergic, cholinergic, and GABAergic neurotransmitter systems can be added [1,13,16,18,23,24,25,34,35,43,69,99]. Large doses of vitamin C could be related to an opposite pro-oxidant effect, as previously debated; however, paradoxically, the potential induced oxidative stress in cancer cells and their subsequent death sustain the efficacy of high doses of intravenous vitamin C in cancer therapy [100]. Despite this, it is important to note that the evidence supporting this efficacy is still limited and controversial [101,102,103], and potential side effects need to be considered, including kidney stones and other adverse reactions. Nevertheless, intravenous vitamin C treatment in cancer patients decreased the toxic side effects of chemotherapeutic agents, likely through vitamin C’s anti-oxidant and anti-inflammatory properties, without interfering with their anticancer action [68].

### 3.3. Archaic Association of Vitamin C with Scurvy Prevention, Related Molecular Mechanisms of Co-Factoring Activities for Collagen Synthesis, and Subsequent Positive Effects on Wound Healing and Tissue Remodeling

Vitamin C plays a very well-known and long-time debated role in collagen synthesis and subsequent scurvy prevention [104]. Scurvy is an acute and profound deficiency of vitamin C that can be fatal without medical intervention [16,105]. Scurvy’s molecular mechanism is a reverse of the basic role of vitamin C in collagen synthesis: a reduced function of prolyl hydroxylase and a production of collagen polypeptides lacking hydroxyproline, leading to unstable triple-helical collagen molecules [36].

Scurvy is a disease characterized by altered functions of connective tissues, perifollicular hemorrhages, and defective healing. Currently, it is mostly found in institutionalized patients, drug addicts, and alcoholics that consume food deprived of vitamin C. Individuals with malabsorption disorders, kidney failure, hemodialysis, and peritoneal dialysis are also included in the group at risk for low levels of vitamin C and for developing clinical symptoms of scurvy [36,40,47]. Clinical signs of scurvy usually develop once vitamin C levels drop below 11.4 μmol/L [21,23,31]. It can be prevented by introducing foods containing vitamin C to diets. The minimal dietary intake of vitamin C to prevent scurvy is about 10 mg per day [4,31], which can be easily achieved by drinking 15 mL of lemon juice.

As a cofactor of three groups of enzymes (prolyl-3-hydroxylases, prolyl-4-hydroxylases, and lysyl hydroxylases), vitamin C is involved in collagen biosynthesis through the hydroxylation of proline and lysine residues of procollagen polypeptides [106]. Although the basic reaction of adding the hydroxyl groups to proline or lysine in the collagen molecule is enzymatically performed, the co-factoring role of vitamin C is that of maintaining the iron cofactor in a reduced state at the active sites of the hydroxylases [107]. As a result of hydroxylation reactions, the procollagen polypeptides gain a triple-helical conformation in the cells, followed by their secretion and polymerization to form the final collagen fibers [24,34,36,108,109]. A minimum of 35% of the prolyl residues must be hydroxylated to stabilize the tertiary structure of collagen at normal physiological temperatures [28].

Ascorbic acid is known for its protective barrier function at the skin level through the stimulation of ceramides in the epidermis as a result of its co-factoring activity for prolyl and lysyl hydroxylation [36,108]. Photo-protection at this level is recognized after topical application, protecting keratinocytes from the damage produced by ultraviolet A (UVA) [36].

Subsequent positive effects of vitamin C on wound healing and bone remodeling are due to the presence of collagen in the organic matrix [13], which is associated with a decrease in the expression of pro-inflammatory mediators, as well as an increase in the expression of wound healing mediators [21]. For example, improved growth and increased synthesis of the extracellular matrix proteins collagen type I (osteonectin and osteocalcin) were reported when increased vitamin C concentrations were added in cultures of primary bovine osteoblasts (up to 200 μg/mL) [110].

When the effect on human dermal fibroblast cultures was tested for a stable vitamin C derivative named magnesium L-ascorbic acid 2-phosphate (AA2P), an increase in dermal fibroblast number and a better organization of the basement membrane zone were observed, as well as an increased expression of genes associated with DNA replication and repair [108]. However, vitamin C is widely recognized as a promoting factor of wound healing and tissue remodeling through increasing collagen gene expression in fibroblasts, as well as promoting fibroblasts proliferation and migration. Moreover, vitamin C influences the activity of some major actors at the wound site in terms of the initial migration of neutrophils for site sterilization and the final process of apoptotic neutrophil clearance by macrophages [21].

Bone disorders in vertebrate organisms (e.g., spontaneous fracturing, impaired bone growth, and impaired bone healing) were associated with a deficiency in the physiological levels of vitamin C, confirming the risk of osteoporosis and fractures resulting from deficient vitamin C intake or decreased serum levels [111].

Vitamin C intervenes in the transformation of vitamin D (synthesized in the skin or absorbed from the intestine) into its active metabolites: 25-hydroxycholecalciferol (25-OH-D3) and 1,25 dihydroxycholecalciferol (1,25(OH)_2_D3). Doses of 150 mg/day i.v. vitamin C for 10 days in 10 human subjects (age range 55–71) promoted the synthesis of 1,25(OH)_2_D3, the active metabolite of vitamin D3 obtained in the mitochondria of the kidney cortical cells from the 25-OH-D3 precursor through the action of the 25(OH)D-1-alpha-hydroxylase enzyme. However, doses of 1000 mg/day acted as inhibitors in this mechanism [112], which is important in the context of vitamin D’s role in bone health, as well as in muscles and the immune system.

### 3.4. Vitamin C and Its Enzymatic Cofactor Activities in Various Metabolisms

Vitamin C is required in vivo as a cofactor for enzymes involved in various processes of biosynthesis (e.g., of carnitine and catecholamine norepinephrine), peptide amidation, and tyrosine catabolism.

(i) Vitamin C is an enzymatic cofactor for carnitine synthesis: Carnitine is an essential cofactor in the transport of long-chain fatty acids into mitochondria in order to produce ATP via beta-oxidation. Although current results are contradictory regarding the essentiality of vitamin C in the biosynthesis of carnitine [113], it remains an important cofactor for the activities of two enzymes (6-N-trimethyllysine dioxygenase and gamma-butyrobetaine dioxygenase) involved in the carnitine biosynthetic pathway, which has long been considered essential in this process [114].
(ii) Vitamin C is an enzymatic cofactor for catecholamine norepinephrine synthesis: The highest concentrations of vitamin C in the body are found in brain and neuroendocrine tissues, such as adrenal [38,69,115]. Vitamin C is a cofactor in the biosynthesis of norepinephrine, firstly in a step mediated by tyrosine hydroxylase, and of tyrosine hydroxylation into 3,4 dihydroxy–l-phenylalanine (L-DOPA) metabolite, followed by the conversion of dopamine (formed after decarboxylation of L-DOPA by aromatic amino acid decarboxylase) to norepinephrine by dopamine beta-hydroxylase [21,116,117,118].
(iii) Vitamin C is an enzymatic cofactor for peptide amidation: Vitamin C acts as a cofactor for peptidylglycine alpha-amidating mono-oxygenase (a copper- and ascorbate-dependent type I membrane protein), the only known enzyme able to catalyze the reaction of amidation of the terminal carboxyl (C-terminal α-amidation) as the final and essential step in the biosynthesis of neuropeptides and peptide hormones [21,119,120,121].
(iv) Vitamin C is an enzymatic cofactor for tyrosine metabolism: Vitamin C is a cofactor of 4-hydroxyphenylpyruvate dioxygenase, a vitamin-C-dependent dioxygenase with a ferrous ion in the active site. This enzyme is involved in tyrosine catabolism, catalyzing the conversion of 4-hydroxyphenylpyruvate to homogentisate (2,5-dihydroxyphenylacetate) [4] through decarboxylation, substituent migration, and aromatic oxygenation in a single catalytic cycle [122]. The final products of tyrosine degradation are fumarate and acetyl coenzyme A (acetyl-CoA), with both playing important roles in energy production [123].

### 3.5. Vitamin C Is an Enzymatic Cofactor That Plays a Role in Gene Transcription

A different mechanism by which vitamin C acts as an enzymatic cofactor for collagen synthesis is related to its role in the upregulation, subsequent stabilization, and prolonged half-life of the resulting transcripts of genes of two types of collagen (I and III) present in skin. Therefore, an increased procollagen *m*RNA level depends on an increased transcription of the genes of collagen I and III upregulated by vitamin C co-factoring of carboxy- and amino-procollagen proteinases and lysosyloxidase [36].

In addition to its function of synthesis, and the export and deposition of the mature collagen process (starting with the hydroxylation of proline and lysine residues), vitamin C also assists other prolyl hydroxylases in the hydroxylation of hypoxia-inducible factor 1α (HIF-1α)1 (HIF-Proline dioxygenase, also named HIF-Prolyl-hydroxylase). HIF-1 is a transcription factor responsible for the cellular response to low-oxygen conditions. HIF-1 acts on target genes that control diverse cellular pathways, including transport of metal ions (e.g., iron transport) and glucose (for glycolysis), cell survival, angiogenesis, vasomotor regulation, and matrix and barrier functions [13,24,47,69,115]. Considering the role of vitamin C in the downregulation of hypoxia-inducible factor and its subsequent regulation of many genes responsible for tumor growth, as well as neutrophil function and apoptosis, the vitamin-C-dependent inhibition of the HIF pathway may provide alternative or additional approaches for controlling tumor progression, infections, and inflammation. Therefore, low vitamin C levels are expected to limit the tumor growth process by compromising the synthesis of collagen, which is required for maintaining both normal vascular function and that of tumor angiogenesis (by means of which the cancerous tissue is provided with metabolic substrates, growth factors, and oxygen) [47].

### 3.6. Vitamin C Is an Enzymatic Cofactor That Plays a Role in Epigenetic Regulation

The role of vitamin C in adrenal steroidogenesis was reported by Bruno [13]. By increasing DNA demethylation and transcription of pro-myelinating genes, vitamin C regulates and sustains the Schwann cells to myelinate axons of the peripheral nervous system. Its action is completed by collagen helices, which stabilize the formation of basal lamina necessary for myelination [124].

Vitamin C is also known as a cofactor for the methylcytosine dioxygenases, particularly the ten-eleven translocation (TET) enzymes, which are involved in DNA and histone demethylation processes [34]. The DNA methylation process is a heritable epigenetic modification, a common denominator in transcriptional repression, genome imprinting, X-inactivation, and carcinogenesis. The reverse process of DNA demethylation is mediated by TET enzymes, which act to convert 5-methylcytosine (5mC) to 5-hydroxymethylcytosine (5hmC). Their activity is enhanced by the addition of vitamin C (to mouse embryonic stem cells), leading to increases in 5hmC content [125]. In vitamin C deficiencies, alterations in the methylation–demethylation dynamics of DNA and histones are described, and these subsequently contribute to phenotypic alterations or even diseases, such as cancer [34,115]. Ramezankhali et al. [126] described vitamin C as a downregulator of TET1 gene expression. The TET1 gene is a DNA demethylating enzyme based on the reversing conversion of 5-methyl cytosine to unmodified cytosine. As a result of its downregulation effect, a negative impact of vitamin C as a reprogramming enhancer of human and mouse somatic cells was established, with a subsequent negative tumor-suppressive effect in human breast cancer cells [126].

Processes of DNA demethylation co-factored by vitamin C were reported for induced pluripotent stem cells (iPSCs). Although the demethylation of DNA firstly occurs only for pluripotency markers, in a further step, widespread DNA demethylation is reasonable [127].

## 4. Endogenous Vitamin C: A General Frame of Interrupted Biosynthesis Pathway 

The genetic basis of vitamin C synthesis is well understood in organisms that can produce it. The pathway involves several enzymes, each encoded by a specific gene. Mutations in these genes can lead to a loss of function and, consequently, an inability to synthesize vitamin C. Understanding the genetic basis of vitamin C synthesis can provide insights into the evolution of metabolic pathways and the factors that influence the genetic diversity of species.

Vitamin C is a highly soluble carbohydrate-like compound synthesized in a large variety of organisms, including plants and some animal species. Yeasts produce D-Erythroascorbate, a C_5_ analog of ASC, while plants and animal species able to synthesize their own ASC use it to enzymatically convert a final substrate into L-ascorbic acid [5,6,27,128].

**Figure 6:**
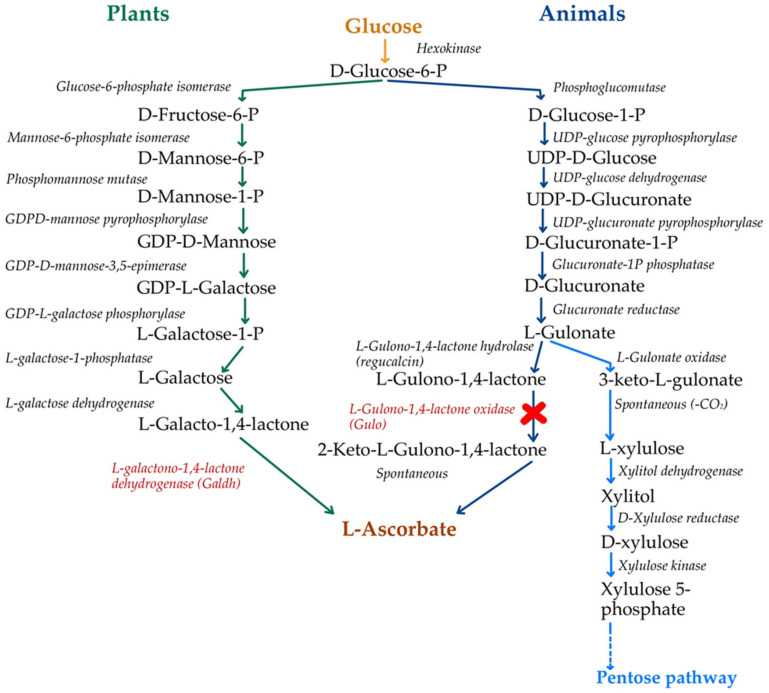
A depiction of the general biochemical pathways of L-ascorbate biosynthesis in plants and animals, with a key step interrupted in some animal species (for plants, adapted from [27,129,130,131,132], while for animals, adapted from [130,132,135,136]).

The Smirnoff–Wheeler pathway, in which vitamin C is synthesized from D-Mannose and L-Galactose (D-Mannose/L-Galactose pathway), represents the major route of vitamin C biosynthesis in plants [27] (Figure 6). The other three routes of vitamin C biosynthesis in plants include the gulose, myoinositol, and galacturonate pathways [7,34,129,130,131,132]. However, the best-understood one remains the L-Galactose pathway, with a control exerted on ascorbate concentration through the transcriptional regulation of the key genes encoding GDP-D-mannose-3,5-epimerase and GDP-L-galactose phosphorylase [133]. The ASC biosynthesis in the Smirnoff–Wheeler pathway is preceded by a last step of enzymatic conversion of L-Galacto-1,4-lactone into L-ascorbate. The involved enzyme, L-galactono-1,4-lactone dehydrogenase (Galdh), allows plants and algae to synthesize vitamin C without also producing reactive oxygen species. This is different from animals, in which ascorbate biosynthesis via Gulo seemed to be associated with the H_2_O_2_ byproduct in Endoplasmic Reticulum lumen [104]; also differently from animals, relatively few transporters are responsible for ASC transport from the site of its synthesis (the inner mitochondrial membrane), whereas the transport of DHA in plants uses glucose transporters [5,27].

The biosynthesis of vitamin C is absent in some fishes, present in the kidneys of amphibians and reptiles, but absent in their liver, and present in the livers of mammals who have kept the ability to produce it, but absent in their kidneys [134]. A majority of animals produce relatively high levels of ascorbic acid from glucose in liver via the glucuronic acid pathway (Figure 6) [15,55,130,132,135,136]. In animal species unable to produce their own ascorbate, the inactivation of synthesis of the Gulo enzyme is often assumed as a result of selective pressure and a mechanism of protection against the resulting H_2_O_2_ byproduct. However, Gulo and Galdh have a mutually exclusive distribution, supported among eukaryotes by one of two evolutionary scenarios, e.g., either an ancient gene duplication in the last common eukaryote ancestor followed by a differential loss or a lateral gene transfer of a novel gene, followed by a functional replacement of the ancestral gene [104].

## 5. Molecular Mechanisms in Vitamin C Transport, Homeostatic Distribution, and Recycling

Three mechanisms mediate the human body’s ascorbate concentrations, with studies of ascorbate ingestion based on dose concentration data showing the following: (i) the intestinal absorption of ingested vitamin; (ii) its accumulation in tissues; and (iii) its renal reabsorption and excretion [135]. Once dietary vitamin C reaches the intestine lumen, it is absorbed (as ASC and DHA) and released into the bloodstream using a concentration-dependent absorption mechanism. More than 95% of circulating vitamin C in plasma is as an unbound ascorbate to plasma proteins. After the subsequent oxidation of ASC, the resulting DHA can be either taken up by erythrocytes to be further recycled into ASC (available now to be released again to the bloodstream) or be distributed and absorbed by tissues and their component cells through molecularly mediated transport [31]. After distribution, plasma levels will be lower than intracellular levels; the extracellular levels, although they may be higher than the plasma levels, still closely reflect the concentrations found at the plasma level. Excretion of vitamin C is achieved mainly through urine, with only 1% through feces. Through the urine, vitamin C is mostly excreted as oxalate, and to a lesser extent as metabolized ascorbic acid, 2,3-diketo-L-gulonate (2,3-DKG), or as DHA [4].

Adrenal glands, ovaries, brain, pituitary gland, liver, spleen, blood cells (but not erythrocytes), and extracellular fluid surrounding the lung and eye are the most concentrated in vitamin C in humans [37,60]. However, high levels of vitamin C in neurons and endocrine tissues, such as the adrenal and pituitary glands, seem to be related to the co-factoring role of vitamin C in the synthesis of hormones and neuro-mediators [4].

If taking into account two forms of ascorbic acid, two mechanisms of cycling vitamin C at the cellular level are known [36]: (i) ASC and (ii) DHA [38,137]. A third mechanism of passive diffusion at the low pH of the gastrointestinal tract can be included for the non-ionized form of vitamin C at this level or for the erythorbic acid isomer [4].

**Figure 7:**
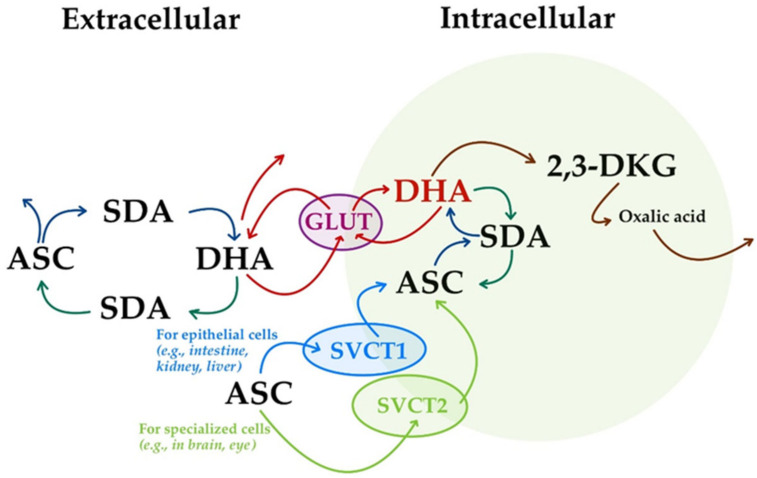
A simplified depiction of vitamin C metabolism. **ASC**—ascorbate; **SDA**—semidehydroascorbate; **DHA**—dehydroascorbate; **2,3-DKG**—2,3-diketo-L-gulonate; **SVCT1, SVCT2**—sodium-dependent vitamin C transporters; **GLUT**—glucose transporter (original graphic representation based on the information described above).

(i) The transport of ASC through the body involves sodium-dependent vitamin C transporters (SVCTs). Two sodium-dependent vitamin C transporters were reported, namely, SVCT1 and SVCT2 [18,37,46,115,138]. They are now considered as the primary means of ASC homeostasis systemic control [22], being crucial players in the process of ASC being taken up by cells in unidirectional mediated transport [4]. The majority of ASC is transported by SVCT1 in epithelial cells (e.g., intestine, kidney, and liver), while the remainder is transported by SVCT2 in specialized cells (e.g., brain and eye) (Figure 7) [38].

Sodium-dependent vitamin C transporter type 1 (SVCT1; encoded by the *SLC23A1* gene in humans, and *svct1* and *slc23a1* in animals) is mostly located in apical brush border membranes of intestinal and renal tubular cells and mediates processes of ASC absorption and reabsorption at this level [69,139,140]. In fact, the role of the SVCT1 transporter in the reabsorption of ASC from the urinary tract is vital [4], and it is also important in perinatal survival and the regulation of vitamin C biosynthesis, as was demonstrated in mice. Although the *scl23a1* -/- individuals were associated with an increased excretion of the ASC fraction (of up to 18-fold), there was an approximate rate of 45% perinatal mortality for both heterozygous and knockout pups born from *slc23a1* -/- dams, with a lower plasma ASC concentration in dams and pups, and these effects were prevented by ASC supplementation during pregnancy [135].

Although the urinary loss of ascorbate decreases concentrations in plasma and in most tissues, this was not observed in the brain. Sodium-dependent vitamin C transporter type 2 (SVCT2; encoded by *SLC23A2* gene in humans, and *svct2* and *slc23a2* in animals) is found in the cells of most other tissues. It is responsible for the transport of ASC into multiple organs from either the blood or the cerebrospinal fluid [140]. SVCT2 was reported as an ASC transporter in the brain [69], and it is critical for maintaining vitamin C levels in fetal and placental tissues, with a lack of SVCT2 and the subsequent low vitamin C levels being associated with fetal death, oxidative stress, and cell death in mice null for SVCT2 (SVCT2−/−) that survived the gestation period. In this last case, significantly lower vitamin C levels were quantified in the placenta, cortex, and lung, but not in the liver, which is their site of vitamin C synthesis. Severe hemorrhages in their cortex were also reported, as well as hemorrhages in the brain stem accompanied by cell loss [140].

The human SVCT1 (K_M_ range: 65–237 μM) is encoded by the human *SVCT1* gene, which has a 1797 bp open reading frame encoding a 598 amino acid polypeptide, while human SVCT2 (K_M_ range: 8–62 μM) is encoded by the human *SVCT2* gene, whose open reading frame is of 1953 bp, encoding a 650 amino acid polypeptide. The *SLC23A1* locus is located on human chromosome 5q31.2-31.3 and contains 15/16 exons, spanning about 17.3 kb. A total of 1440 single-nucleotide polymorphism (SNP) variations are known, of which 294 are located in the coding region (187 missense, 91 synonymous, 11 frameshift, and 4 insertions). The *SLC23A2* locus is located on human chromosome 20p12.2-12.3 and contains 17 exons, spanning about 160 kb. A total of 8165 SNP variations are known, of which 262 are located in the coding region (138 missense, 120 synonymous, and 4 frameshift). The genetic patterns of both human *SLC23A1* and *SLC23A2* genes were described to share common intron/exon borders and to have related coding sequences, but with differences in gene sizes, for example, 17.3 kb compared with 160 kb, respectively (about 10-fold difference in size) [31,137,141].

The human SVCTs share an identity of 65% [31], while a significant amino acid identity similarity of 60% was reported between human SVCT1 and SVCT2 and their mouse homologs [137]. The gene for human SVCT1 is located in an area that also includes the loci for Charcot–Marie–Tooth disease (with autosomal recessive inheritance) and limb-girdle muscular dystrophy type 1A. The gene for human SVCT2 is located in an area that contains the locus for another neurological disorder, Hallervorden–Spatz disease, which is characterized by progressive dementia and brain iron accumulation. Both host human chromosomes 5 and 20, are involved in tumor genetic changes, but neither of them nor the corresponding mouse loci of the vitamin C transporter genes (located on chromosome 18 and 2, respectively) are involved in osteoporosis or other bone defects [142].

Such sodium-dependent ASC transport proteins are also known in rats, namely, rSVCT1 and rSVCT2. In contrast, the ASC pathway in humans is dependent on vitamin C dietary intake [18,20]. An age-related decline in *SVCT1* expression in rat liver cells was reported, with a subsequent possibility of occurring in humans; therefore, there is a need for dietary supplementation with vitamin C in the elderly [16]. However, compared to the average plasma vitamin C levels of 40–60 μM in adults, those found in children between the ages of 6 and 11 are higher, gradually decreasing in adulthood [4].

The expression of *SVCT1* and *SVCT2* genes is inversely regulated by intracellular ASC levels, as the depletion of ASC results in increased hepati*c SVCT1* and *SVCT2* expression. In addition, the expression of *SVCT2* is regulated by nitric oxide in an NF-κB-dependent manner, while a mechanism of low-density lipoprotein intervenes in endothelial cells [20]. Being an important factor for assuring vitamin C levels in the brain, the expression of *SVCT2* is increased after vascular brain injury [4].

Five alleles of single-nucleotide polymorphisms (SNPs) in the *SLC23A1* gene were reported as associated with lowered serum ascorbic acid concentrations, although some of their genotypes showed mixed results in various studies. For example, in a modeled dose vs. plasma concentration study, the A772G allele of *SLC23A1* was associated with the deficient vitamin C phenotype regardless of the levels of vitamin C intake (up to 2400 mg/day) [31]. In the case of the *SLC23A2* gene, there are ~2200 identified SNPs expected to influence the tissue levels of ASC [31]. In 2018, Granger and Eck [105] showed only three SNPs as associated with lowered serum ascorbic acid concentrations [105]. Some variations in *SLC23A1* were reviewed as associated with an increased risk of Crohn’s disease, non-Hodgkin lymphoma, preterm delivery, and aggressive periodontitis, while some variations in *SLX23A2* were shown as being associated with an increased/decreased risk of gastric cancer, increased risk of bladder cancer, and decreased risk of colorectal adenocarcinoma, HPV16-positive head and neck cancer, non-Hodgkin lymphoma, chronic lymphocytic leukemia, preterm delivery open-angle glaucoma, and acute coronary syndrome in women [4]. However, polymorphisms in the genes encoding SVCTs can significantly influence vitamin C homeostasis, affecting susceptibility to various diseases with important implications for human health. Studies showed that individuals with certain SVCT polymorphisms experience reduced vitamin C absorption, leading to lower plasma and tissue vitamin C levels, and altered vitamin C distribution, affecting the availability of vitamin C in specific organs. As health implications, variations in vitamin C status due to SVCT polymorphisms may influence susceptibility to oxidative stress, impairing anti-oxidant defense mechanisms and increasing the risk of oxidative damage and chronic diseases, such as cardiovascular, cancer, or age-related macular degeneration [143,144,145].

(ii) The oxidized form of ASC, DHA, is transported in the body using glucose transporters (GLUTs) (Figure 7) [37,115]. Thus, ASC is oxidized at the extracellular level to form DHA, and is subsequently transported intracellularly using GLUT1 (*SLC2A1* in humans) and GLUT3 (*SLC2A3* in humans) glucose transporter isoforms [18]. The isoform GLUT4 (*SLC2A4* in humans) seems to be used only for insulin-sensitive tissues [20,36,141]. However, unlike unidirectional transport facilitated by SVCTs, the GLUTs mediate bidirectional DHA-facilitated transport (Figure 7) [4].

At the intracellular level, DHA is rapidly reduced to ASC by NADH- and NADPH-dependent reductases, as well as by reduced glutathione (GSH) [137]. Sharing the same transporters as glucose, GLUT-mediated transport of DHA is competitively inhibited by glucose. Therefore, excess glucose in plasma or intestine can decrease the absorption of DHA [4,31]. This fact is important in hyperglycemic conditions caused by diabetes that require clinical administration of vitamin C [16]. A genome-wide association study showed that there is no association between genetically predicted plasma vitamin C levels and type 2 diabetes and, therefore, no strong evidence to suggest the use of vitamin C supplementation for type 2 diabetes prevention [146]. Nevertheless, potential glycemic control improvements in people with type 2 diabetes were reported in a short-term study that considered the use of vitamin C supplementation [147].

In total, 4 of 14 glucose transporters (GLUT 1-4) were found by Lindblad et al. [31] to be involved in the intracellularly facilitated diffusion of DHA. They have a general structure that includes 12 transmembrane domains and a length of ~500 amino acids; GLUT 1-3 were reported to be located on the basolateral membrane (GLUT 1), on the apical brush border membrane (GLUT 3), and on both sides of the membrane (GLUT 2) [31].

A complex model of five DHA transporters in humans was described by Shaghaghi et al. [141]: GLUT1 (*SLC2A1*), GLUT2 (*SLC2A2*), GLUT3 (*SLC2A3*), GLUT4 (*SLC2A4*), and GLUT8 (*SLC2A8*). All of them are members of the *SLC2A* solute carrier gene family, which encodes glucose transporter (GLUT) proteins. The GLUTs express tissue and cell affinities for DHA transport: GLUT1 is expressed in an extensive variety of cells throughout the body, with a particularly high expression in endothelial and epithelial-like barriers of the brain, peripheral nerve, eye, placenta, and lactating mammary gland; GLUT2 is mainly expressed in the brain, spleen, kidney, pancreas, liver, and basolateral membranes of intestinal epithelial cells; GLUT3 is expressed particularly in the brain, neurons, and intestinal epithelial cells; GLUT4 is mainly found in adipose tissues as well as skeletal and cardiac muscle cells; and GLUT8 is expressed in the testis, blastocyst, brain, muscle, and adipose tissues. GLUT1 has the highest probability of mitochondrial localization; vitamin C enters mitochondria in its oxidized state via GLUT-1 [141].

Of particular interest is the increased expression of GLUT1 on erythrocytes as a compensatory mechanism for vitamin recycling in mammals that cannot synthesize vitamin C. In fact, GLUT1, a glucose transporter that also transports DHA (the oxidized form of vitamin C), allows, through its increased expression, an efficient uptake of DHA from the extracellular environment, thus facilitating its intracellular reduction back to ascorbate. Being constantly exposed to oxidative stress, erythrocytes have significantly higher levels of GLUT1, thus supporting the vital role of ascorbate in protecting them from oxidative damage [142].

## 6. Fluctuating Gene Activity and the Evolutionary Puzzle of *GULO* Loss of Function

L-gulono-1,4-lactone oxidase (Gulo) represents a membrane-bound oxidoreductase enzyme of the Endoplasmic Reticulum that aerobically catalyzes the conversion of L-Gulono-1,4-lactone to L-ascorbate, with H_2_O_2_ as a byproduct (Equation (1) [94]). Unlike its plant counterpart, L-Galactono-1,4-lactone dehydrogenase (L-Galactono-γ-lactone dehydrogenase, Galdh), which acts specifically on L-Galactono-1,4-lactone, Gulo acts preferentially on L-Gulono-1,4-lactone, but also on L-Galactono-, D-Mannoro, and D-Altrono-1,4-lactone [5,148].

L-gulono-1,4-lactone + O_2_ → L-ascorbate + H_2_O_2_ (1)

Gulo is 50.6 kDa protein in humans, encoded by the rest of the primordial gene located on chromosome 8 (8p21). Other *pseudo GULO genes (Ψ GULO)* are located on chromosomes 14 in pigs, 15 in rats, 2 in rabbits, and 2 in turkeys. The dog, cat, and giant panda possess an additional intronless *GULO* retro pseudogene (e.g., in dogs, the *GULO* pseudogene and its retro pseudogene are located on chromosomes and 25 and 33, respectively). *GULO* orthologues of mammalian *GULO*-neighboring genes were scattered on chromosomes 20 and 17 in zebrafish, a teleost fish with a complete loss of the *GULO* gene from its genome [5,19,148].

The fluctuating activity of the *GULO* gene, dependent on dietary vitamin C intake, requirements for energy, oxidation, or individual development, was reported in rabbits. In this species, the activity of the *GULO* gene is higher in winter than in summer. Rabbits fed indoors showed decreased *GULO* gene activity compared to those fed outdoors. In sturgeons and other vertebrate species, higher levels of *GULO* expression were reported during their embryonic stages of development than in any other period of their life [19].

In humans, non-human primates, guinea pigs, dogs, some but not all bats, and some bird species, the absence of synthesis of L-gulono-1,4-lactone oxidase is an inborn error of metabolism caused by various changes in the structure of the original *GULO* gene [13,18,20,22,23,28,68,149]. On the other hand, two bat species, namely, *Rousettus leschenaultia* and *Hipposideros armiger*, are thought to have regained a functional *GULO* gene, although discussions on the lack of functionality of the produced L-gulono-1,4-lactone oxidase in the *Chiroptera* order are still carried out [150]. Although pika *(Ochotona princeps)* was previously reported with a functional *GULO* gene [19], recent findings contradict this assumption [150].

The ability to synthesize vitamin C developed as species evolved, with vertebrates transitioning from water to land. Some fishes have lost their ability to synthesize vitamin C, but not amphibians, reptiles, and phylogenetically older species of birds. Their ability to produce vitamin C in the kidneys was a necessary adaptation to counteract the aggressiveness of atmospheric oxygen towards pulmonary tissue [151].

**Figure 8:**
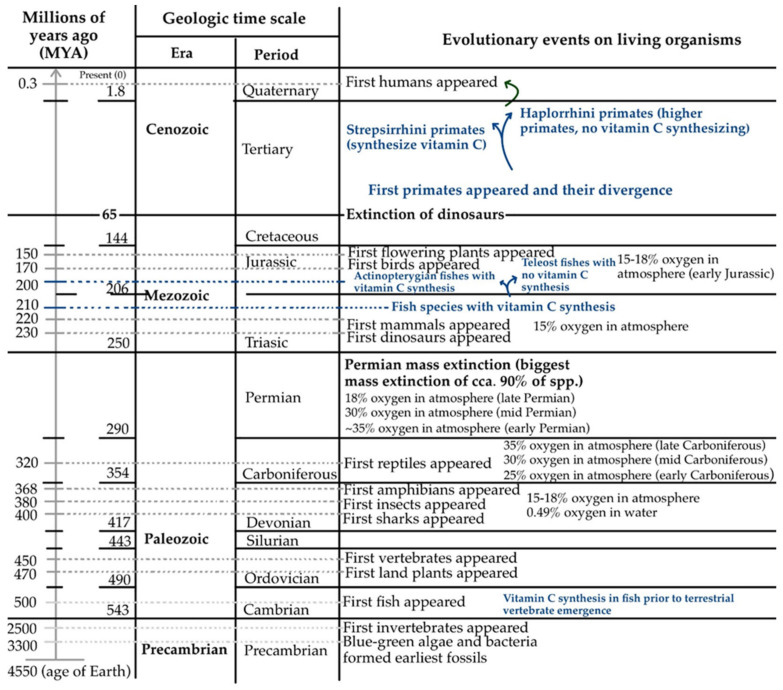
The main evolutionary events that influenced vitamin C synthesis (original graphic representation based on information presented in [19,134,150,152,153]).

The first amphibians and reptiles appeared on Earth a long time after the first fishes, in periods of atmospheric oxygen concentration instability, reaching hyperoxia conditions (25–35% in the Carboniferous period). This atmospheric oxygen overload came after a period of 15 to 18% oxygen concentration in the atmosphere (in the late Devonian period), and lasted not only through the Carboniferous period but also into the Permian (Figure 8). These prolonged hyperoxia conditions seemed to be fatal to many living tetrapods and contributed to what we know today as the largest Permian mass extinction. Even so, many species survived, most likely due to the expression of the *GULO* gene and their own synthesis of vitamin C in the body as a protective factor against the toxicity of oxygen [134].

The site of vitamin C biosynthesis in amphibians and reptiles is the kidney, an organ that offers a much lower synthesis yield (16–20 times lower) compared to the liver—the site of vitamin C production in mammalian species capable of vitamin C synthesis [115,134]. This was an adaptive mechanism to their increased requirements during vitamin C synthesis, in which the larger size of liver was associated with higher levels of biosynthesized vitamin C [151]. However, towards the end of the Permian and then in the Mesozoic period, including the Triassic and later the Jurassic, the atmospheric oxygen level dropped to values of 15–18% found much earlier, such as in the Paleozoic Devonian (Figure 8). This made the anti-oxidant fighting mechanism through vitamin C synthesis less effective, and perhaps even harmful as a consequence of the residual products obtained on the biosynthesis path starting from glucose. As a consequence, the synthesis activity of the enzyme L-gulono-1,4-lactone oxidase has decreased alongside phylogenetic evolution, with evolutionary loss suffered in some species of higher animals [1,13,18,20,22,23,134]. Considering the potentially toxic H_2_O_2_ resulting from the vitamin C biosynthesis pathway (Equation (1)), the loss of function of the *GULO* gene could therefore be an evolutionary improvement for the control of redox homeostasis [34]. Moreover, it is believed that during the evolution of primates, uric acid might have taken over the anti-oxidant function of vitamin C, as there are records of these species regarding their inability to break down uric acid and to synthesize vitamin C [19,27,39].

The *GULO* gene was deactivated at different moments in time: 61 million years ago for *Haplorrhini* primates (suborder of higher primates, which includes humans, tarsiers, monkeys, and apes) and 14 million years ago for the guinea pig. The lower primates *Strepsirrhini* (including lemurs, lorises, and Galagoes) still retained their ability to synthesize vitamin C [16,19,39,150,152]. When *Haplorrhini* primates diverged from the *Strepsirrhini*, the *Strepsirrhini* kept an intact *GULO* gene structure, while all species belonging to the higher suborder *Haplorrhini* have *GULO* pseudogenes in their genome *(GULOP)* (Figure 8), with similar mutation patterns significantly degenerating the 440-amino-acid-long sequence [150]. As a result, higher primates, including humans, orangutans, chimpanzees, gibbons, and macaques, are characterized by their lack of ability to synthesize vitamin C to satisfy their own needs. They are not the only deficient vertebrate species that depend on exogenous vitamin C. Guinea pigs and some but not all bats are also characterized by the loss of function of the *GULO* gene [19]. Notably, although there is no synthesized L-gulono-1,4-lactone oxidase in teleost fishes (rainbow trout, the Japanese medaka, the common carp, and zebrafish), several ancestral actinopterygian fish species retained the ability to synthesize vitamin C. However, inactivating mutations of the *GULO* gene seemed to occur in teleost fish 200–210 MYA, after the divergence of these two lineages [152].

But why did vertebrates lose the ability to synthesize vitamin C? This is a longstanding question in evolutionary biology. While most animals can produce their own vitamin C, vertebrates (including humans) cannot. The prevailing theory suggests that this loss was a trade-off during their evolution [153]. Here is a breakdown:

(i) A relatively high metabolic cost: While the exact metabolic cost can vary depending on factors such as species, metabolic rate, and dietary conditions, it is generally considered to be relatively high considering the required energy and many enzymes involved. First of all, the vitamin C biosynthesis pathway starts with glucose [128], a simple sugar derived from food. Converting glucose into intermediates necessary for vitamin C synthesis consumes energy, primarily in the form of ATP (adenosine triphosphate). Furthermore, there are several enzyme-mediated reactions with different enzymes involved, each catalyzing a specific reaction. Some catalytic enzymes require cofactors (such as NADPH—nicotinamide adenine dinucleotide phosphate) to function and whose regeneration is also energy-consuming [154]. All of these have several evolutionary implications regarding adaptation to reduce the metabolic burden associated with vitamin C synthesis, freeing up energy for other essential functions in many vertebrates, including humans [155].
(ii) Dietary availability: As vertebrates evolved and diversified, many species adapted to diets rich in vitamin-C-containing fruits and vegetables, which made the metabolic cost of internal synthesis less necessary and reduced the selective pressure to maintain the ability to synthesize vitamin C internally. This led to an evolutionary adaptation that allowed these species to reduce their metabolic burden and focus on other essential functions [156].
(iii) Genetic drift: Over time, genetic drift (random changes in gene frequencies) may have led to the loss of the gene responsible for vitamin C synthesis in certain vertebrate lineages. This could have occurred especially in species with abundant dietary sources of vitamin C. Genetic drift is a random process that can cause changes in gene frequencies within a population, especially in small populations. It is a key mechanism of evolution, and it likely played a role in the loss of the vitamin C synthesis gene in vertebrates [157].

Small populations sizes, neutral mutations, genetic bottleneck, or the founder effect independently or in combination may have contributed to the loss of function of the vitamin C gene. If the population of a vertebrate species was small at some point in its evolutionary history, random fluctuations in genes frequencies will have had a considerable impact [158]; otherwise, the gene for vitamin C synthesis, for example, could have become less frequent due to chance. If a mutation occurred in the gene for vitamin C synthesis that rendered it non-functional while insignificantly affecting the organism’s survival or reproduction, this neutral mutation, despite not having a significant effect on an organism’s fitness, can constitute a reservoir of new phenotypes in the long-term evolution of an involved population [159]. A genetic bottleneck occurs when a population undergoes a drastic reduction in size, often due to factors like natural disasters or disease. This can lead to a loss of genetic diversity [160], including the gene for vitamin C synthesis. The founder effect occurs when a small group of individuals colonizes a new habitat. This can result in a loss of genetic diversity [161], including the gene for vitamin C synthesis, if the founders do not carry the gene. However, in the case of the vitamin C synthesis gene, it is likely that a combination of these factors gradually contributed to its reduced frequency until it was eventually lost in vertebrates.

(iv) Additional hypotheses can also be taken into account, such as selection for other factors or the intervention of environmental factors. For example, it is possible that the gene involved in vitamin C synthesis was repurposed for other functions, making its loss advantageous; while there is no definitive evidence that this has happened in vertebrates, it is a possibility that scientists are exploring. Repurposing of genes often occurs when a gene’s original function becomes less important or when a new function offers a selective advantage [150]. Hypothetical examples of how the vitamin C synthesis gene might have been repurposed take into account the possibility that the enzymes involved in vitamin C synthesis were readapted to participate in other metabolic pathways, such as those involved in energy production or detoxification. Alternatively, the gene products may have evolved new functions in cellular signaling, allowing them to regulate various cellular processes, or the synthesizing gene could have been co-opted to play a role in gene expression, such as controlling the activity of other genes [162]. However, it is important to note that these are speculative examples. More research is needed to determine if the vitamin C synthesis gene has indeed been repurposed in vertebrates and, if so, what its new functions might be.

Regarding the environmental factors, a wide range could have been involved in the loss of function of synthesis. For example, changes in the environment and the availability of vitamin-C-rich foods may have influenced the loss of the gene. Environmental changes (including not only climate change, but also habitat changes, dietary adaptation, or competition and predation) could have had a significant impact on the evolution of species, including the loss or retention of specific genes [162]. Shifts in climate patterns could have affected the distribution and abundance of vitamin-C-rich plants. For example, periods of drought or extreme cold might have reduced the availability of certain fruits and vegetables, making it more advantageous for vertebrates to rely on internal synthesis of vitamin C. Changes in habitat, such as deforestation or the emergence of new ecosystems, could have altered the availability of vitamin-C-rich foods. For instance, the colonization of new environments with limited vitamin C resources might have favored individuals with the ability to synthesize vitamin C internally. As vertebrates evolved and diversified, they adapted to different diets. Some species may have developed specialized digestive systems or behaviors that allowed them to extract vitamin C from a wider range of foods. This could have reduced the selective pressure to maintain the vitamin C synthesis gene. Changes in competitive pressures or predation could have influenced the dietary habits of vertebrates. For example, if a species faced competition for limited vitamin-C-rich resources, it might have evolved to rely on other food sources or to synthesize vitamin C internally [19].

By understanding the environmental factors that may have influenced the loss of the vitamin C synthesis gene, scientists can gain insights into the evolutionary history of vertebrates and the complex interplay between genetics and the environment. While the exact reasons for the loss of vitamin C synthesis in vertebrates remain a subject of ongoing research, the trade-off between metabolic cost and dietary availability provides a compelling explanation. For example, the dog is one of the animal species that has lost its ability to synthesize its own vitamin C, likely due to a genetic mutation that occurred in its evolutionary history related to dietary adaptations [163]. Although dogs are historically considered carnivores, their diet is actually omnivorous. This may be more pronounced after their distinct evolution from wolves, although the latter also has a diet that is occasionally associated with the consumption of berries and other vitamin-C-rich fruits [164,165]. Moreover, although meat itself does not contain significant amounts of vitamin C, the organs of prey animals, particularly the liver, can provide small amounts. However, this loss of ability to synthesize vitamin C seems to have provided an evolutionary advantage regarding adaptation to different environments and food availability. Furthermore, this flexibility may have contributed to their successful domestication and global spread [166].

## 7. Gene to Pseudogene Transformation: A Journey into Genetic Silence

The transformation of a gene into a pseudogene is a complex process driven by various genetic mechanisms. While pseudogenes may appear to be evolutionary relics, they can play important roles in gene regulation, genome evolution, and human disease [167,168].

A pseudogene is a segment of DNA that resembles a gene but has lost its ability to code for a functional protein. This transformation from a functional gene to a pseudogene is a fascinating process that can occur through various mechanisms, including its duplication and divergence, accumulation of mutations, or the loss of its regulatory elements. During meiosis, misalignment of chromosomes can lead to an unequal exchange of genetic material, a process known as unequal crossing over, which can result in gene duplication—when a copy of a gene is created. Another mechanism that might be involved here is retrotransposition, when a processed *m*RNA transcript is reverse-transcribed into DNA and inserted into a new genomic location. This new copy, lacking regulatory elements and introns, is often non-functional. Regarding the possibility of the accumulation of mutations, single-nucleotide changes or point mutations can disrupt the reading frame, introduce stop codons, or alter the amino acid sequence, leading to a non-functional protein. Moreover, deletions and insertions are large-scale changes that can also disrupt a gene’s structure and function. Mutations in the promoter region can prevent transcription initiation, while disruptions in enhancer sequences can reduce gene expression [150,168,169,170].

While pseudogenes may seem like evolutionary leftovers, they serve several important molecular functions, such as those related to genetic redundancy and regulatory RNA. Thus, some pseudogenes may provide a backup copy of a gene, allowing for potential future adaptation, and certain pseudogenes can be transcribed into non-coding RNAs that regulate gene expression [168,171,172].

Studying pseudogenes can provide clues about the evolutionary history of genes and genomes. Moreover, although pseudogenes are generally considered non-functional, they can sometimes contribute to disease [173]. For example, gene conversion events between a functional gene and a pseudogene can introduce deleterious mutations into the functional gene [174].

Regarding the *GULO* gene, this was inactivated in primates through a series of genetic mutations. These mutations accumulated over millions of years and ultimately rendered the gene non-functional. As a result, primates, including humans, lost the ability to synthesize vitamin C and must obtain it from their diet. All of the above-presented mechanisms of a gene transformation into a pseudogene could also be found in the case of the *GULO* gene. However, it is important to note that the exact sequence of events leading to the inactivation of the *GULO* gene is still being studied. However, the combined effect of these genetic mechanisms ultimately resulted in the loss of vitamin C synthesis in primates [175].

**Figure 9:**
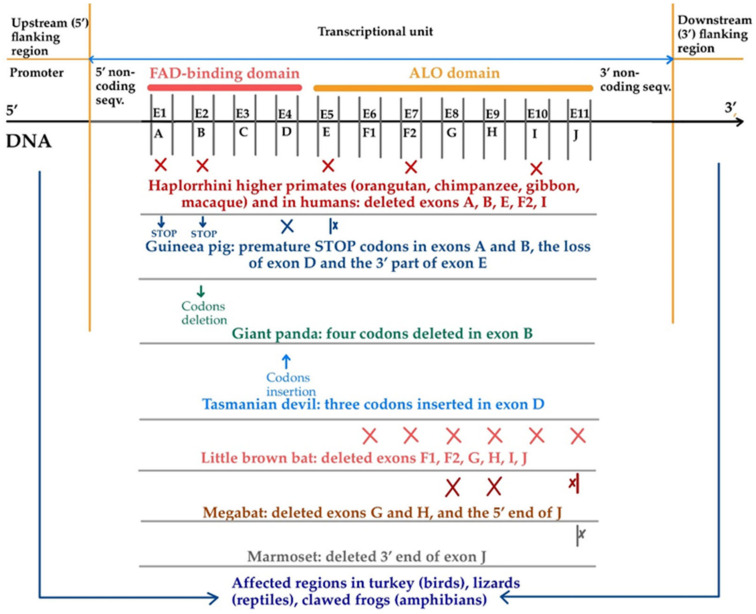
One view on molecular variations in *GULO* gene pseudogenization (adapted from [19]).

Investigations on vertebrate *GULO* gene structure showed a range of 64–95% for sequence similarities at the level of encoded amino acids. The gene structure is highly conserved, traditionally including 11 exons noted as A, B, C, D, E, F1, F2, G, H, I, and J. Exon F was subdivided into F1 and F2 during the evolution of the animal GULO gene. Exons A, B, C, and D form the FAD-binding domain, in which the cofactor FAD is covalently bound by a histidine residue. The rest of the seven conserved exons constitute another essential domain named ALO (D-arabinono-1,4-lactone oxidase), in which ligands are bound by several residues (Figure 9) [19].

On the other hand, the rat *GULO* gene, which is ~23 kilobase pairs (kbp) in length, consists of 12 exons and 11 introns, and is now considered a general guide to exon presence and absence in degraded *GULO* genes in other mammals. Compared to this structure, the guinea pig *GULO* gene has two affected exons, with the loss of exon D and the 3′ part of exon E, accompanied by premature stop codons in exons A and B (Figure 9) [5,19,134,152,176].

The innovative research of Mansueto and Good [150] showed a different structure for the mammalian *GULO* gene. From a total of 12 exons, the first exon contains a 5′ untranslated region and the ATG start codon, while the subsequent II-V exons and a part of exon VI code for the FAD-binding domain, with a part of exon VI and exons VII-XII coding for the ALO domain. Related to this newly proposed structure, it seems that the *Haplorhini GULOP* sequence only retained the identity of exons IV, V, VII, IX, X, and XII, while the rest of them are degenerated [155]. Moreover, in *Haplorhini* primates, exons IV, IX, and X were described as generally affected by insertions, with exons VII, IX, and X described as affected by deletions; exon IX is also affected by deletions in Old World Monkeys of *Haplorhini* primates, and exon VII is also affected by insertions in *Rhesus macaque* (Old World Monkeys of *Haplorhini* primates). Exon V is affected by deletions in *Rhesus macaque* and drill (Old World Monkeys of *Haplorhini* primates) [150]. In total, 18 unique and conserved individual substitutions were reported for Old World Monkeys (excepting *Rhesus macaque*, with 44 substitutions described), while there were just 11 in hominids. However, the number of substitutions per site seemed to be greater at the node of the phylogeny when the *Haplorhini* and *Strepsirrhini* initially split [150]. When discussing the *Rodent* order, a larger range in substitutions per site was reported, especially in the *Muridae* and *Caviidae* families [155]. A hypothesis of chromosomal inversion as a causative factor for the pseudogenization of the *GULO* gene was tested for *Haplorhini* primates, but was not totally accepted, although this kind of chromosomal rearrangement may increase the variability of nucleotides within populations and across species [150].

## 8. The Role of Vitamin C Gene Production and Pseudogene Transformation in Human Health

In the field of medicine, understanding the genetic factors that influence human health is crucial for developing effective treatments and interventions [177]. One such genetic factor that has garnered significant attention is the production of the vitamin C gene and the potential impact of pseudogene transformation on human health.

Unlike most animals, humans are unable to synthesize vitamin C due to the lack of a functional gene encoding for the production of the enzyme L-gulono-1,4-lactone oxidase, which is essential for vitamin C synthesis. This genetic deficiency has long been a subject of interest in the medical community, as it raises questions about the potential health implications associated with the inability to produce vitamin C endogenously [32,178].

Recent research has shed light on the role of pseudogenes in potentially compensating for the missing functional gene responsible for vitamin C production in humans. Pseudogenes are non-functional copies of genes that have lost their protein-coding ability due to mutations or other genetic alterations. While pseudogenes were originally thought to be genetic “junk”, recent studies have suggested that they may have regulatory functions and could potentially act as modulators of gene expression [150,167,168,169,170].

The hypothesis of pseudogene transformation in human health posits that certain pseudogenes may undergo functionalization or regulatory activities that compensate for the absence of the functional gene involved in vitamin C production. This raises intriguing questions about the potential adaptive mechanisms that have evolved in humans to cope with the genetic deficiency related to vitamin C synthesis [155].

Understanding the interplay between the missing vitamin C gene and the potential role of pseudogenes in human health has significant implications for clinical practice and public health. Firstly, elucidating the mechanisms by which pseudogenes may influence vitamin C production could lead to the development of targeted therapies or interventions aimed at modulating pseudogene activity to enhance vitamin C levels in individuals with genetic deficiencies. Furthermore, gaining insights into the genetic adaptations related to vitamin C production could inform personalized medicine approaches, allowing healthcare professionals to tailor interventions based on an individual’s genetic makeup. This could pave the way for more precise and effective treatments for conditions associated with vitamin C deficiency, such as scurvy and certain types of anemia [179].

Scurvy, a disease historically associated with vitamin C deficiency, is now targeted by focusing on the potential influence of genetic factors that modulate an individual’s susceptibility to this disease, including *GULO* pseudogene polymorphism. By analyzing the genetic variations in this pseudogene, researchers aim to elucidate the interplay between these polymorphisms and an individual’s risk of developing scurvy [148]. This line of investigation holds promise for uncovering novel genetic markers that could aid in identifying individuals with heightened susceptibility to scurvy, thereby enabling targeted interventions and preventive measures. Moreover, understanding the impact of vitamin C pseudogene polymorphism in scurvy is not only pertinent from a clinical perspective but also holds implications for public health initiatives. By discerning the genetic underpinnings of scurvy, healthcare professionals can devise more effective strategies for population-wide vitamin C supplementation and dietary recommendations. Additionally, insights into genetic predisposition to scurvy can inform screening protocols aimed at identifying at-risk individuals and facilitating early interventions to mitigate the progression of the disease [180].

Among genetic polymorphisms, the vitamin C pseudogene polymorphism has also emerged as a subject of interest, particularly regarding its potential association with anemia. Anemia, characterized by a deficiency in red blood cells or hemoglobin, remains a prevalent global health concern [155]. The etiology of anemia is multifaceted, encompassing nutritional deficiencies, chronic diseases, and genetic predispositions. Studies have indicated a putative association between certain allelic variants of the vitamin C pseudogene and anemia susceptibility [19]. Notably, individuals carrying specific polymorphic variants may exhibit altered red blood cell parameters, including hemoglobin levels and red blood cell counts. Furthermore, research has suggested that these polymorphic variations may modulate oxidative stress pathways, potentially exacerbating the pathophysiology of anemia [181].

The presence of the vitamin C pseudogene in the human genome has sparked curiosity among researchers regarding its potential impact on the aging process. Some studies have suggested that the inactivation of the *GULO* gene and the subsequent reliance on dietary vitamin C may have broader implications for aging and age-related diseases [182]. One proposed mechanism for the link between the vitamin C pseudogene and aging involves the role of vitamin C as an anti-oxidant. Anti-oxidants play a crucial role in protecting cells from oxidative stress, which is known to contribute to aging and age-related diseases [183]. As a potent anti-oxidant, vitamin C helps neutralize free radicals and reduce oxidative damage in the body. Therefore, the reliance on dietary vitamin C due to the inactivated *GULO* gene may impact the body’s ability to combat oxidative stress effectively, potentially contributing to the aging process. Moreover, the presence of the vitamin C pseudogene has also been associated with certain age-related conditions [184]. For instance, studies have explored the potential connection between vitamin C deficiency, which may be exacerbated by the inactivated *GULO* gene, and age-related diseases such as cardiovascular disease, cognitive decline, and cataracts [185,186,187].

## 9. Conclusions

Ascorbic acid participates in many biological processes, enzyme activities, cell signaling pathways, immune defense systems, and collagen synthesis. It is vital to prevent and cure scurvy and the oxidative damage that accompanies subclinical ascorbic acid deficiency, which may be further involved in the aging process, degenerative disorders, or cancer. Humans and non-human primates, guinea pigs, some bats, and several bird species are unable to synthesize vitamin C, mainly through the loss of function of their *GULO* genes. The evolutionary cause for this loss remains controversial. Arguments include that organisms evolved protection against potentially toxic H_2_O_2_ resulting from vitamin C biosynthesis, that the dietary intake of vitamin C suppressed its own synthesis in a mechanism of positive selection for suppressing the accumulation of toxic byproducts, or that there were mechanisms of neutral mutation and neutral evolution, as *GULO* gene losses and gains of function have been reported in some bats and passerine birds.

In the field of medicine, the study of genetic variations in *GULO* pseudogenes and their impact on disease susceptibility and progression is of paramount importance. Understanding the specific mechanisms through which the inactivated *GULO* gene may contribute to scurvy or anemia susceptibility or influence aging processes and age-related diseases could potentially pave the way for novel interventions and specific therapeutic approaches. In addition to its clinical implications, research into vitamin C gene production and pseudogene transformation also contributes to our broader understanding of human and animal evolution and the process of genetic diversity. Unraveling the genetic mechanisms that underlie human adaptation to dietary and environmental factors provides valuable insights into the complex interplay between genetics and health.

## Figures and Tables

**Table 1:** Normal ascorbate levels in various human tissues, human blood plasma, and selected cells, as well as in some human fluids [33].

Tissue	Mean Value (µmol/L)
Brain	800–900
Lungs	400
Skeletal muscle	200–300
Spleen, liver, pancreas	600–900
Adrenals	1700–2300
Kidneys	300–900
Saliva	0.6
Gastric juice	136
Urine	200
Blood plasma	50
Neutrophil	1350
Monocyte	3100
Lymphocyte	3800
Platelet	2790
Red blood cells	45
